# Subtly encouraging more deliberate decisions: using a forcing technique and population stereotype to investigate free will

**DOI:** 10.1007/s00426-020-01350-z

**Published:** 2020-05-14

**Authors:** Alice Pailhès, Gustav Kuhn

**Affiliations:** Psychology Department, Goldsmiths, University of London, New Cross, London, SE14 6NW UK

## Abstract

Magicians’ forcing techniques allow them to covertly influence spectators’ choices. We used a type of force (Position Force) to investigate whether explicitly informing people that they are making a decision results in more deliberate decisions. The magician placed four face-down cards on the table in a horizontal row, after which the spectator was asked to select a card by pushing it forward. According to magicians and position effects literature, people should be more likely to choose a card in the third position from their left, because it can be easily reached. We manipulated whether participants were reminded that they were making a decision (explicit choice) or not (implicit choice) when asked to select one of the cards. Two experiments confirmed the efficiency of the Position Force—52% of participants chose the target card. Explicitly informing participants of the decision impairs the success of the force, leading to a more deliberate choice. A range of awareness measures illustrates that participants were unaware of their stereotypical behaviours. Participants who chose the target card significantly underestimated the number of people who would have chosen the same card, and felt as free as the participants who chose another card. Finally, we tested an embodied-cognition idea, but our data suggest that different ways of holding an object do not affect the level of self-control they have over their actions. Results are discussed in terms of theoretical implications regarding free will, Wegner’s apparent mental causation, choice blindness and reachability effects.

## Introduction

We like the feeling of being in control of our thoughts and our actions, and yet many of our behaviours are systematically influenced by external and internal factors (Ariely, 2008; Bargh & Chartrand, 1999; Bargh, Chen, & Burrows, 1996; Loewenstein, 1996). Likewise, our thoughts are often less unique than we intuitively believe them to be, and research on population stereotypes illustrates that most people will choose or think about similar things or objects when asked to make a decision (French, 1992; Grimmer & White, 1986; Marks & Kammann, 1980). Understanding the external factors that influence our behaviours may help individuals make more informed and freer choices (Appourchaux, 2014).

Baumeister suggested that free will is predominantly associated with cognitive processes involving conscious and controlled activity (i.e. System 2), rather than the nonconscious and automatic processes associated with System 1 (Baer, Kaufman, & Baumeister, 2008; Kahneman et al., 2002). Accordingly, a more useful view of free will is to think in terms of autoregulation and self-control mechanisms, a perspective that allows us to take advantage of the parameters influencing our thoughts and actions during our day to day lives. Magicians are masters at deception and creating the illusion of conscious will, and they use a wide range of forcing techniques, to give spectators the illusion that they freely and consciously chose a card, which in reality is predetermined by the conjurer (Kuhn, Amlani, & Rensink, 2008). This paper uses a forcing technique to investigate whether explicitly informing people that they are making a decision leads to a more deliberate decision.

### Forcing Techniques

Forcing refers to conjuring techniques which allow magicians to covertly influence a spectator’s choice or its outcome (Pailhès & Kuhn, 2019; Pailhès & Kuhn, submitted). These techniques are often used to create the illusion of precognition or mind reading and magicians have extensive real-world experience in manipulating the decisions people make. Back in 1894, Alfred Binet investigated magicians’ deceptive craft scientifically, and he observed that conjurers exploit spectators’ “laziness” without them becoming aware of it (Binet, 1894). In other words, conjurers intuitively try to manipulate the spectator into using more automatic cognitive processes, which are easily exploited to trick the mind. He further noted that magicians often use circumstantial influences to push a person to act in a predictable way. Nowadays, we refer to these processes as automatic behaviours, which often rely on heuristics, or a System 1 type of thinking (Kahneman, 2011; Kahneman et al., 2002). By observing conjurers performing tricks, Binet noted that if you are presented with three different objects, one alongside the other, most people choose the middle one. He also points out that this is probably due to the ease by which people execute certain grasping actions. Likewise, he noted that when people are presented with a sheet of paper that has been divided into 16 equal size squares, and they are asked to draw a dot into one of them, most people will choose the middle squares. As he writes, “there is therefore a kind of attraction exerted by the centre of the figure. Probably also because they provide more convenience to the hand.” (Binet, 1894, p.150/151). Magicians frequently exploit these types of cognitive heuristics and population stereotypes to force a decision (Annemann, 1940; Banachek, 2002; Jones, 1994). Magicians’ real-world experience and expertise in performing these tricks for large audiences have allowed them to identify psychological factors that enhance the possibility of the spectator selecting the forced item.

Several other papers have investigated forces that rely on different techniques and it is likely that spectators simply choose the easiest option. The “Classic Force” relies on the timing in which the magician is handling the deck of cards while asking the spectator to pick a card (Shalom et al., 2013). Shalom et al. showed that most people pick the card which is subtly handled by the magician who physically restricts the choice. Olson, Amlani, Raz, & Rensink (2015) investigated the “Visual Riffle Force” in which spectators are asked to visually select a card when the magician flips through the deck in front of their eyes—most spectators choose the card which is the most visually salient. Both forces have high success rates and showed that participants felt free even when they chose the target card.

**Fig. 1:**
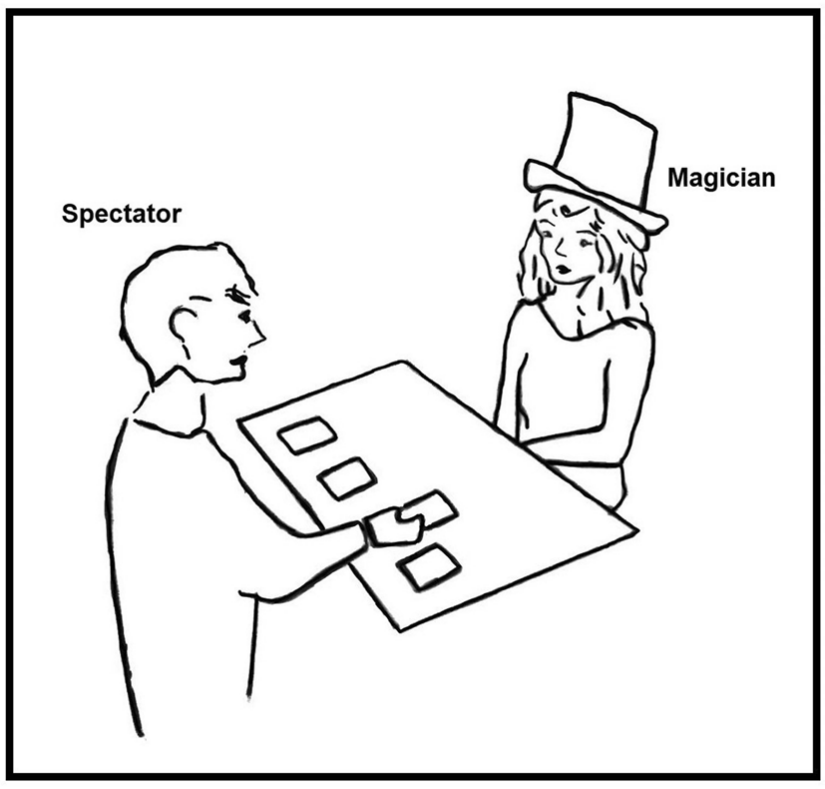
Representation of the Position Force in which the spectator/participant selects the third card from his left by pushing it towards the magician/experimenter

Magicians have developed a large assortment of forcing techniques that rely on a wide range of cognitive processes (Pailhès & Kuhn, 2019). In this paper, we examine a forcing technique that relies on population stereotypes: the Position Force. This technique is based on the observation that people’s choices for random objects are influenced by the object’s physical position. According to the magic literature, people will be inclined to select the card that is the easiest to reach in the row (Banachek, 2002; Binet, 1894). This force is most commonly used with five playing cards (Banachek, 2002), but we decided to investigate the force with four cards to compare the results to forcing from our research program: here, the magician places four cards on the table in a horizontal row, after which the spectator is asked to select a card, by physically touching it. Results from an online survey on 91 magicians showed that most of them (68%) think that when we present four cards in a row on a table to spectators, the majority will choose the third card from their left. Their mean estimation of the percentage of people who would choose this target card was 57% of the spectators (SD = 15.9). Indeed, a recently published study from our laboratory using the Position Force found that 60% of the participants select the third card from their left while feeling free for their choice and underestimating the proportion of people who would select the same card (Kuhn, Pailhès, & Lan, 2020) (Fig. 1).

Moreover, research in other domains suggests that people’s choices are influenced by the physical positioning of an object.

### Position effects

Nisbett and Wilson showed in 1977 that when presented with four identical pairs of stockings, people tend to prefer the far-right one (Nisbett & Wilson, 1977). Nowadays, consumer psychology (Chae & Hoegg, 2013), and nudge techniques (Dayan & Bar-Hillel, 2011) often rely on manipulating an object’s physical positioning with the intention of influencing people’s behaviour and choices. For example, people are more likely to choose an item, such as food (Kim, Hwang, Park, Lee, & Park, 2019), if it is positioned in a specific location, and this can be used to lead people towards healthier choices (Bucher et al., 2016). There are however some discrepancies about the exact way in which positioning affect people’s choices, with studies showing both edge advantage and aversion. Bar-Hillel suggests that these inconsistencies result from different choice characteristics, such as whether it is interactive or not (Bar-Hillel, 2015a). Accordingly, a choice is interactive when the payoff for someone’s decision is affected by another interested person. For example, in a game such as rock, paper, scissors, each player’s choice payoff is determined by the joint choices of both players. A further factor involves the amount of cognitive processing a choice requires to figure it out. Situations in which all items are evidently identical (such as the back of playing cards) fall into the category of choices that neither require processing nor interaction. In this case, we observe that people present an edge aversion rather than edge advantage (Bar-Hillel, 2015a, b).

Indeed, when presented with a selection of similar options, or identical items, individuals tend to choose items located in the middle position rather than those located at the edges (Christenfeld, 1995). This effect has been found with a range of items. For example, participants prefer middle items and avoid items located at the extremes when choosing among a row of arbitrary symbols, a toilet paper roll within a stall, a bathroom stall, and when picking products from shelves in supermarkets. The principle ruling these effects is thought to be based on a minimal mental effort (Christenfeld, 1995; Shaw, Bergen, Brown, & Gallagher, 2000). Indeed, research showed that when participants are asked to choose between similar highlighters, survey papers, or seats, they reliably prefer the middle items (Shaw et al., 2000). Bar-Hillel (2015a, b) notes that in such situations, it is not necessarily mental effort, but also physical one which is at play. The author further suggests that in these type of tasks, middle items are more reachable than those at the ends, because they are closer to the participants. Indeed, her principle of reachability dovetails this idea in that when all things are equal, people prefer objects that can be reached more easily. Accordingly, when people are presented with a horizontal physical display, their choice will be biased by this reachability principle, which might explain why they favour central items.

This behaviour, using a principle of least effort, is linked to dual-system theories of cognition (Chaiken, Liberman, & Eagly, 1989; Chen, Duckworth, & Chaiken, 1999; Evans & Stanovich, 2013; Kahneman, 2011; Kahneman, Frederick, Kahneman, & Frederick, 2001; Petty & Cacioppo, 1986) which argue that most of the time we use automatic, rapid, stereotyped responses rather than controlled ones (Tomlin, Rand, Ludvig, & Cohen, 2015). Research on the psychology of the self suggests that one of the most important human characteristics is the ability to modify our responses and therefore remove ourselves from effects of situational stimuli (Baumeister & Heatherton, 1996). It has been shown that self-control requires attention and effort (Baumeister, 2002; Baumeister & Heatherton, 1996; Hagger, Wood, Stiff, & Chatzisarantis, 2010) and that one of the main functions of our reflective system is to control thoughts and actions suggested by our automatic, impulsive system (Kahneman, 2011). Our System 1 (automatic type of thinking) is associated with greater use of diverse biases and heuristics, rather than our deliberative, reflective processes (Kahneman et al., 2002). Therefore, encouraging people to reflect before making a decision is expected to lead to lesser use of impulsive behaviours. Although there is some research examining the psychological factors that activate our automatic type of thinking (e.g. cognitive load and time pressure, Baumeister & Heatherton, 1996; Hwang, 1994; Vohs & Heatherton, 2000), less is known about how to activate more deliberate decisions.

This paper seeks to document the Position Force, investigating its success rate and how free participants feel even when they are influenced by the trick. At the same time, we seek to investigate whether it is possible to encourage participants to make more deliberate choices, impairing the success of the force. In Experiment 1, we examine whether a simple change in phrasing, making the choice explicit, can lead to this effect.

## Experiment 1

Experiment 1 aimed to empirically examine how effective the Position Force is in terms of forcing participants to choose a target card, and to investigate whether the nature of the choice affects the extent to which participants choose the predicted item. Participants were either asked to simply push a card towards the experimenter (implicit choice), or they were explicitly asked to choose a card before the physical selection (explicit choice). Previous research shows that deliberative decision-making can be induced by simply framing tasks as decisions rather than intuitive reactions (Small, Loewenstein, & Slovic, 2007; Zhong, 2011). For example, participants were asked to “decide” rather than “to feel” to induce a deliberative decision (Zhong, 2011). Deliberative decisions are thought to lead to less reliance on heuristics and impulsive, automatic behaviours (Boureau, Sokol-Hessner, & Daw, 2015; Kahneman & Frederick, 2002; Stanovich & West, 2000). We therefore predicted that participants would be less likely to choose the target card (i.e. card that could be reached more easily) when they were encouraged to deliberately think about the choice (i.e. explicit choice) rather than when they made the selection implicitly. In line with previous research on the reachability bias (Bar-Hillel, 2015a, b; Bar-Hillel, Peer, & Acquisti, 2014), we predicted that the force would only work for participants who used their right hand to reach for the card, and thus it should be more effective for right-handed participants.

Our second objective was to examine the extent to which participants were aware of the force. To our knowledge, none of the previous studies on position effects and reachability has done this (though see Kuhn et al., 2020). Two key elements make a force successful: participants must select the target object, and this selection must feel free. Therefore, we assessed how free people felt about their choice and their awareness about the bias itself. Since the Position Force is commonly used in the context of a magic performance, we predicted that participants should feel free about their selection and that they are unaware of this behavioural bias.

## Methods

### Participants

One hundred participants (50 females, 50 males) between 18 and 60 years old (*M* = 29.71, SD = 11.65) recruited on Goldsmiths University campus took part in the experiment. Goldsmiths Psychology Department provided ethical approval for the two experiments. Before the experiment and to maximize the power of our results, we ran an a priori power analysis for a Chi-squared test with *w* = 0.30 (moderate effect size), *α* = 0.05, and a power of 0.8. The output required 88 participants and the chosen effect size was based on prior results using the Position Force (Kuhn et al., 2020). We confirm that for both experiments, we report all measures, conditions and data exclusions.

### Procedure

The experimenter/magician sat at one of Goldsmiths’ cafeteria table with the four cards already on the table, all spaced by approximately 5 cm, and positioned on the table in a way which made the row as symmetrical as possible. Participants sat to face the experimenter. Participants were randomly allocated to one of the two selection types (implicit choice or explicit choice) and consent forms presenting the experiment as a study about magic tricks and decision-making were signed. In the implicit choice condition, participants were asked to “push a card toward [the experimenter]”. The procedure for the explicit choice condition was identical with the exception that they were instructed to “choose a card, and then push it toward [her]”.

The experimenter then noted the chosen card and the hand with which the participant pushed the card. The participants were then asked to complete a questionnaire which asked them (1) how free they felt about their choice (from 0, not free at all to 100 completely free), (2) the percentage of people they thought would have chosen the same card as them, and (3) the Dutch Handedness Questionnaire (van Strien, 2003).

## Results and discussion

### Efficiency of the force and main manipulation

**Fig. 2:**
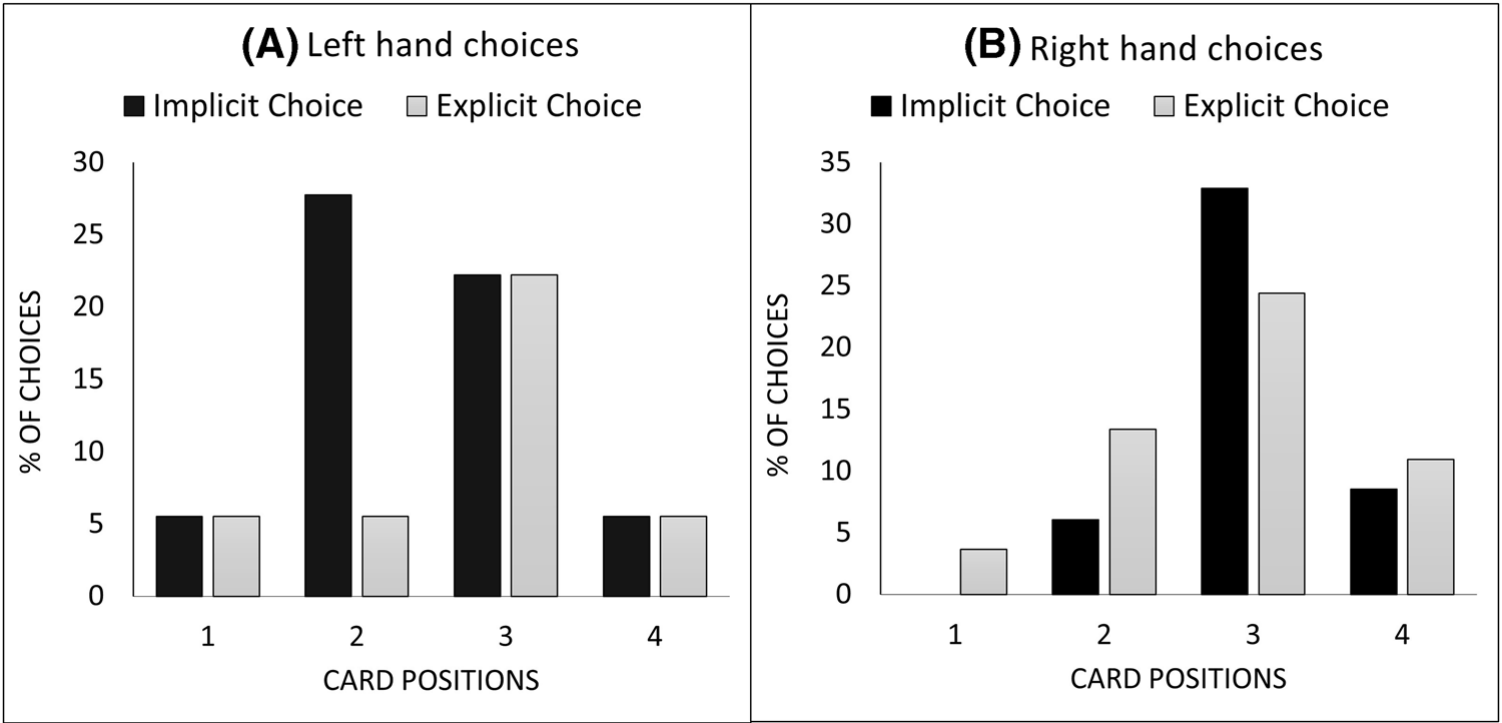
Percentages of choices as a function of the experimental conditions and the hand used to make the selection. **a** The choices made by participants who used their left hand to push the card, **b** for those who used their right hand. Position 1 is the first card from the left of the participants

The first analysis aimed to assess the efficiency of the Position Force and the impact of the nature of the choice on participants’ selection. Figure 2 shows the percentages of participants who chose each of the four cards as a function of the nature of the choice and the hand that was used to make the selection. Eighteen percent of participants used their left hand, compared to 82% who used their right hand. Overall, 55% of the participants chose the target card, which was the most chosen card, significantly more than chance (i.e. 25%) (*X*^2^ (1, *N* = 200) 18.75, *p* < 0.001, *φ* = 0.293). This result very closely matches the mean of magicians’ estimates (57%).

A visual inspection of the graphs illustrates a systematic difference in selections as a function of the hand used to make the selection.^1^ Although the graph highlights clear differences in the success of the force as a function of hand selection, the difference did not reach statistical significance (*X*^2^ (3, *N* = 100) 3.95, *p* = 0.27, *φ* = 0.195). However, since only 18% of the participants used their left hand it is likely that non-significant difference is due to a lack of power. As we expected the force to work only when people used their right hand, we focused the rest of the analyses for the right-handed selection only. Participants in the implicit choice condition were significantly more likely to choose the target card than those in the explicit choice condition (*X*^2^ (1, 82) 4.32, *p* = 0.038, *φ* = 0.224). This suggests that as we predicted, people tend to act in a more deliberate way when they are reminded that they are making a decision.

### Awareness of the force

**Fig. 3:**
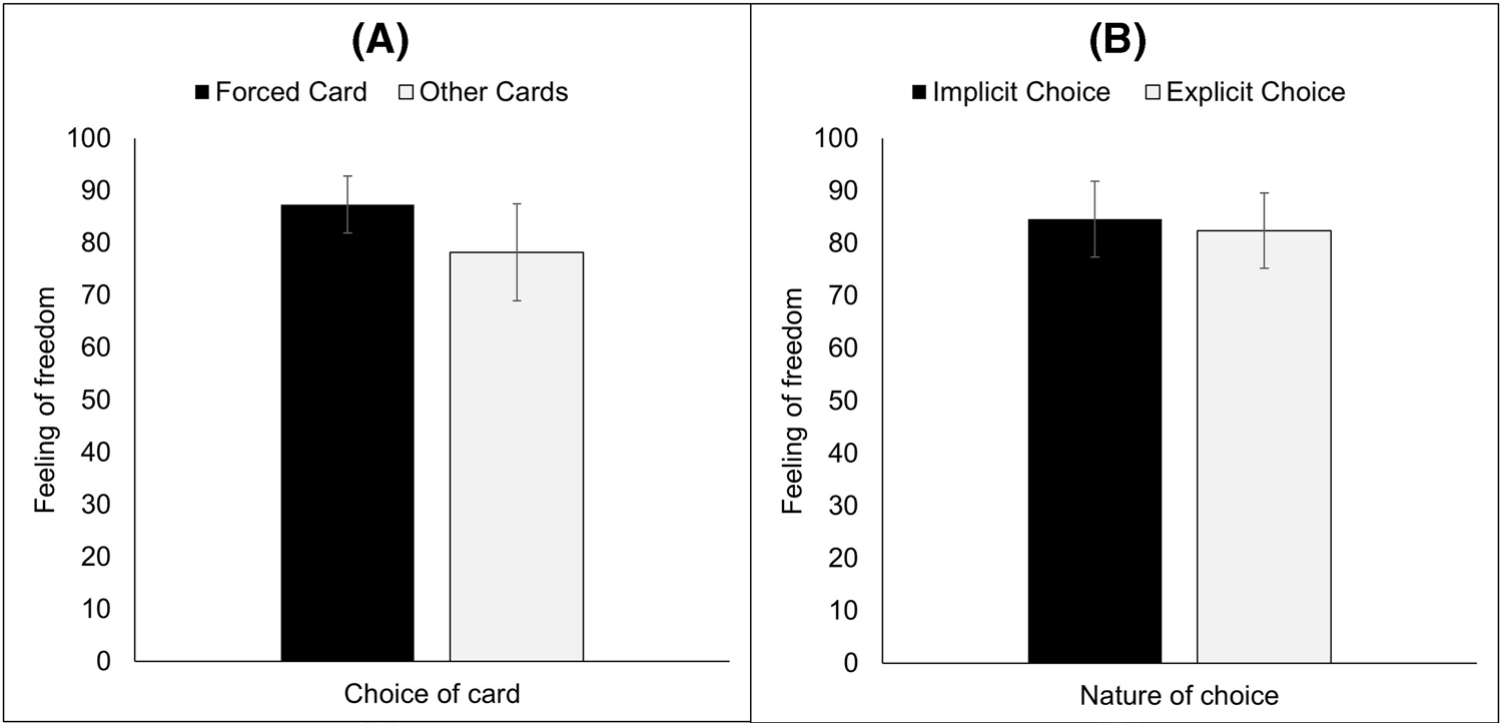
Mean feelings of freedom as a function of whether participants chose the target card or not (**a**) and of the experimental manipulation (**b**). Bars show 95% confidence intervals

Our next analysis examines the impact that the nature of the choice (explicit vs. implicit) and the choice itself (forced or not) has on people’s feeling about how free the choice was. Kruskal–Wallis tests show that neither the choice of card nor the selection method had an impact on participants’ feeling of freedom for their choice (H(1) = 1.77, *p* = 0.18 and H(1) = 0.17, *p* = 0.68, see Fig. 3). This shows that participants are unaware of their bias, as well as a dissociation between their behaviour and their conscious introspection.

**Fig. 4:**
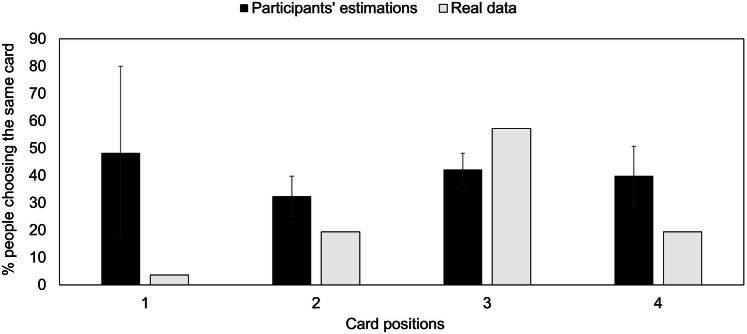
Participants’ estimations of the percentage of people who would have chosen the same card as they did, and the real data from our experiment. 95% confidence interval bars are displayed for participants’ estimations

Next, we examined participants’ metaknowledge of the bias by examining their estimates for the percentage of people who would choose the same card. Kruskal–Wallis test shows that whatever card the participants chose, they did not give different estimations of the percentage of people who would choose the same card as they did (H(3)2.46, *p* = 0.48). Interestingly, participants who chose the target card underestimate the fact that they used a population stereotype, and the other participants overestimate the number of times their card would be chosen (see Fig. 4). This shows again an important dissociation, this time between participants’ behaviour and their evaluation of others’.

These results suggest that the Position Force is effective—a large proportion of our participants chose the target card, while not being aware of their bias. This confirms Wegner’s theory (Wegner, 2002a, b), showing that people tend not to have access to the real causes of their behaviours, which are often unconsciously rooted. Here, most participants’ decision seems to have been guided by the position of the card while they underestimated the number of people who would have made the same decision. A simple change in phrasing negatively impacted the success of the Force. This suggests that participants rely less on automatic/impulsive biases when asked to choose before acting. Handedness also plays a role in this force—the force only worked when people used their right hand. The results confirm previous literature about reachability and edge aversion when presented items are identical, as participants favoured items which were easier to reach according to the hand their used while avoiding the cards at the ends of the row.

## Experiment 2

The second experiment aimed to replicate the results from Experiment 1 and confirm whether explicitly informing people about the choice before their selection would impair people’s stereotypical behaviour. This time, rather than letting people use their preferred hand, we forced them to use their right hand by restricting the use of their left hand. We used this experiment to test a controversial idea in embodied cognition which suggests that the nature in which they are asked to hold an object influences the level of self-control they have over their actions. This idea is based on the observation that people may clench their fists, tense their muscles or grit their teeth when firming willpower, and argues that such actions could also help us firm willpower and consequently improve self-control (Hung & Labroo, 2010; Niedenthal & Barsalou, 2005). Past research on embodied cognition shows that participants’ self-control is enhanced when they firmly grasp an object while making a choice (Hung & Labroo, 2011; Niedenthal & Barsalou, 2005). The explanation behind these findings is that our memories would be composed of multimodal experiences, which also spread throughout our body. One consequence of this would therefore be that bodily actions accompanying thoughts could generate the associated cognitions and influence our behaviours (Briñol & Petty, 2003; Cacioppo, Priester, & Berntson, 1993). If true, it predicts that participants would experience greater self-control, therefore choosing the target card less often when they were asked to firmly grasp a glue stick rather than simply hold it. Finally, we decided to investigate participants’ sense of freedom more thoroughly, using Thompson's 3 components of a free choice (Thompson, Locander, & Pollio, 1990): being deliberate, in control, and free from restriction.

## Methods

### Participants

One hundred participants (59 females, 40 males, 1 non-binary) between 18 and 65 years old (*M* = 30.19, SD = 11.73) recruited on Goldsmiths University campus took part in the experiment.

### Procedure

The experiment took place at the same venue, with the same setting at Goldsmiths University, where the participants were recruited. This time, every participant was asked to hold a glue stick in their left hand. The experimenter either asked them (while doing the gesture herself) to simply hold the glue stick in their open palm or to firmly grasp it between their fingers and their palm.

As in Experiment 1, participants were randomly allocated to one of the selection conditions and either asked to “choose a card and then push it towards [the experimenter]” (explicit choice), or to “push a card towards [the experimenter]” (implicit choice). Participants were then asked to put the glue stick down and answer the paper questionnaire. The questionnaire was composed of 0–100 scale questions about their feeling of freedom (“How free did you feel for your choice?”), its three components (“How restricted did you feel for your choice?” “How impulsive/deliberate did you feel in making your choice?” and “How much control did you feel you had over your choice of card?”), as well as two measures about how firmly and tightly they felt their hand while making the choice to ensure they did tense their muscles more in the self-control condition. Finally, their writing hand, gender and age were also recorded.

## Results and discussion

### Efficiency of the force and main manipulations

**Fig. 5:**
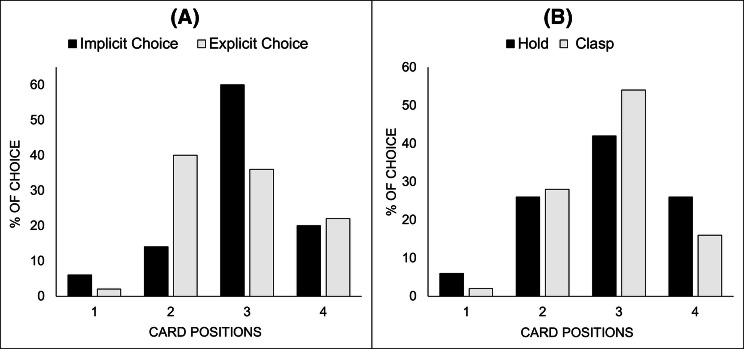
Percentages of choice for each card depending on the choice conditions (**a**) and embodied self-control condition (**b**). Position 1 is the first card from the left of the participants

Our first analysis tested the efficiency of the Position Force and our two main manipulations. Figure 5 shows the percentages of participants who chose each of the four cards as a function of the two experimental manipulations.

Overall, 48/100 chose the target card, which was the most frequently chosen one. Comparing our results to a random distribution (25% choice per card), a Chi-squared showed that our participants chose the target card significantly more often than the others (*X*^2^ (1, 200) 11.41, *p* < 0.001, *φ* = 0.232).

Regarding the experimental conditions, participants chose the target card significantly less often in the explicit choice condition than in the implicit choice condition (36% vs 60% of choices, *X*^2^ (1, 100) 5.77, *p* = 0.016, *φ* = 0.234). This confirms that when participants are forced to use their right hand to make their choice, and therefore when the most convenient card to choose is indeed the forced one, the phrasing of the choice does have an impact on whether or not participants use a stereotypical answer. It appears that simply using the sentence “choose a card, and then push it towards me” rather than just “push a card towards me” subtly make the choice more salient and explicit, therefore activating a more deliberative process in participants’ decision. However, no significant difference was found regarding the effect of embodied self-control (*X*^2^ (1, 100) 1.44, *p* = 0.23, *φ* = 0.119), even though participants did feel their hand muscles were significantly tighter (*W* = 1954, *p* < 0.001) and firmer (*W* = 1980, *p* < 0.001) when they were asked to firmly grasp the glue stick rather than simply hold it. Several explanations seem possible in regard of these null results. First, studies using this type of procedure have suggested that firmly clasping an object could enhance self-regulation and control (e.g. withstand pain, overcome food temptation, consume unpleasant medicines). But we cannot rule out the possibility that this does not apply to the current specific situation. It is also possible that the present study does not necessitate participants to use their self-control to choose a card other than the forced one, and therefore an enhanced self-control would not affect the results. However, embodied cognition theories have also suffered from important criticism regarding their grounding in theoretical background, and several papers have put in doubt the validity of research on the subject (Caramazza, Anzellotti, Strnad, & Lingnau, 2014; Goldinger, Papesh, Barnhart, Hansen, & Hout, 2016; Mahon & Caramazza, 2008) or lack of replication (e.g. Chabris, Heck, Mandart, Benjamin, & Simons, 2019). It has been pointed out that within most experiments on embodied cognition, the expected behaviours tended to be overarching ones (e.g. completing a task), and our study was probably looking for a more specific outcome (Goldinger et al., 2016).

### Feeling of freedom

**Fig. 6:**
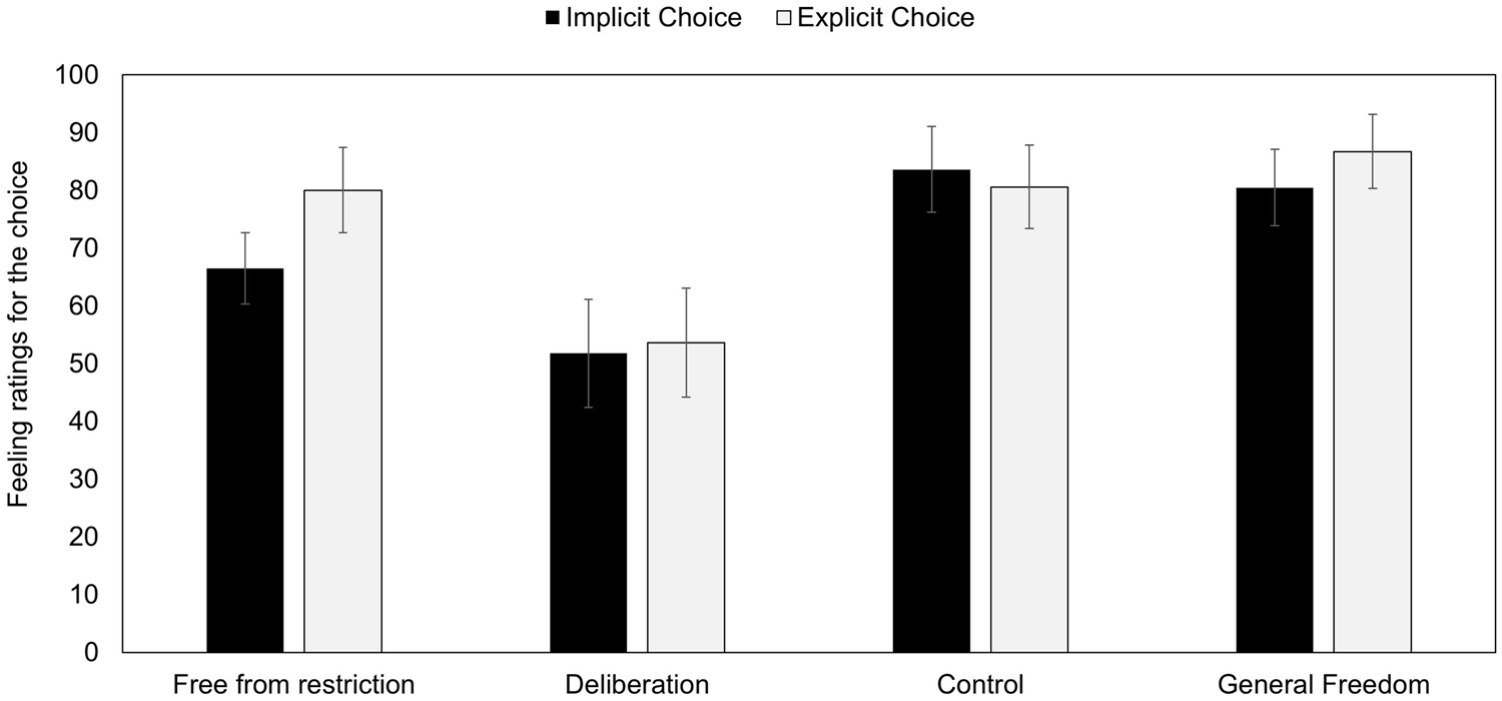
General feeling of freedom and its components as a function of the experimental conditions. Bars are 95% confidence intervals for each condition

First, regarding the overall general sense of freedom, participants felt significantly freer in the explicit choice condition than in the implicit one (*W* = 1540, *p* = 0.034, *r*_pb_ = 0.232, see Fig. 6). No significant difference was found for the embodied self-control variable (*W* = 1330, *p* = 0.56, *r*_pb_ = 0.064). Taking a closer look at the components of the feeling of freedom (Fig. 6), participants felt significantly more free from restrictions when the choice was explicit (*M* = 80.06) rather than implicit (*M* = 66.52, H(1) = 6.63, *p* = 0.01). No other significant result was found regarding either the self-control variable or the other components of freedom (i.e. the feeling of control and deliberation).

The mean of the feelings of control, restriction and deliberation was correlated with the general feeling of freedom (*r*_*s*_ = 0.619, *p* < 0.001, see Table 1). However, the feeling of deliberation did not seem to correlate with those of control, restriction, and general freedom. A calculation of Cronbach’s alpha appeared to be only 0.34 for the three items but went up to 0.60 if the item of the feeling of deliberation was removed. It then appears that contrary to Thompson’s definition (1990), the feeling of deliberation is not a reliable component of people’s general feeling of freedom.

**Table 1:** Spearman correlations with the three components of freedom, their mean and the general sense of freedom

		1.	2.	3.	4.	5.
1.	Deliberation	—				
2.	Control	0.069	—			
3.	Restriction	0.074	0.537***	—		
4..	*M* 1, 2, 3	0.623***	0.690***	0.704***	—	
5.	General freedom	0.137	0.525***	0.684***	0.619***	—

The item for the feeling of restriction was reverse (on a scale from 0 to 100, 100 was completely free from restrictions)

***p < 0.001

Finally, we looked at how the feeling of freedom and its components were linked to participants’ choice of card. Results of the logistic regression indicated that there was no significant association between participants’ feeling of freedom, restriction, control and their choice of cards (*X*^*2*^(95) = 4.93, *p* = 0.295). However, the feeling of deliberation was significantly associated with the participants’ choices (*p* = 0.045). Indeed, the more participants felt their decision was deliberate, the more likely they were to choose another card than the forced one. During debriefings, participants who did not choose the target card typically reported first thinking about taking it and then changing their mind for another card.

In summary, this experiment replicated experiment 1, showing that most participants tend to choose the target card and that the Position Force is extremely effective. We confirmed that the nature of the choice has an impact on whether they choose the target card or make a more deliberate choice and go for another one.

Moreover, the more participants felt their choice was deliberate, the less likely they were to choose the target card. Also, participants felt less restricted and more generally free when they were asked to “choose” a card (explicit choice) rather than simply “push” it (implicit choice). However, the embodied self-control variable did not show to have any impact on any measure.

## General discussion

This paper sought to document the Position Force, as well as investigate whether it was possible to lead people to act more deliberately when making a simple decision. For this, we used a subtle change in the phrasing of the choice, making it either explicit or implicit (Experiment 1 and 2), as well as a controversial idea in embodied cognition (Experiment 2).

### Position Force’s efficiency and choice variable

Both experiments confirmed that the Position Force is efficient and replicate previous results (Kuhn, Pailhès & Lan 2020), with an overall 52% of participants choosing the target card (the third one from participants’ left). These results closely match the mean of magicians’ estimates (57%) and demonstrate that magicians’ intuition about the effectiveness of the force is pretty accurate and precise. Our results further show that a position effect influences people’s choice, and they clearly illustrate an edge aversion effect, which dovetails previous findings that have used identical items (Bar-Hillel, 2015a, b; Christenfeld, 1995). It is interesting to note that some other related forcing techniques might rely on this principle as well. Dai Vernon’s five cards force is thought to rely on reverse psychology and five cards are placed in a horizontal row with the target card located fourth from the left. In this force, five cards are carefully chosen, namely the king of hearts, seven of clubs, ace of diamonds, four of hearts and nine of diamonds (from left to right). The spectator is primed to be suspicious as the magician insists the selection must be a free choice and points out that the ace is in the middle and the seven is the only black card. These statements are thought to eliminate these two cards as they were mentioned. The two last cards are situated at the end of the row, and the king is the only picture card which is suggested to make it suspicious. As stated by Banachek (2002), the four of hearts is more likely to be chosen as it is not at the end of the spread and is in the fourth position. It would be interesting to investigate whether this force truly relies on reverse psychology, or simply on the position of the card—again seemingly the most reachable one.

The two experiments also showed that asking participants to make an explicit decision impairs the success of the Force. When participants were asked to choose a card and then push it rather than simply push it, they chose the target card less often. These results suggest that the subtle change in the presentation of the choice resulted in less automatic, and more deliberate choices. Therefore, it seems that making a choice explicit leads to a less automatic, impulsive decision: a more deliberate one.

### Awareness of the bias

Experiment 1 investigated participants’ awareness of their bias, asking them to estimate what percentage of people would have chosen the same card as they did in the same situation. The results show that participants’ choice of card had no impact on their estimation. Across the four different types of choices (the four cards), participants estimated that between 33 and 43% of other people would choose the same card as theirs. Participants who chose the target card underestimated the fact that their choice was a population stereotype, their mean estimation being 40%, compared to the 55% of participants who chose the identical item. However, participants who did not choose the target card, gave overestimations of the frequency of other people’s choices. The mean of their estimations across the three cards was 38% compared 15% who chose these cards. This adds to previous literature in choice blindness and highlights a dissociation between our behaviour and our conscious introspection (Hall, Johansson, Tärning, Sikström, & Deutgen, 2010; Hall et al., 2013; McLaughlin & Somerville, 2013). As Wegner noted, the actual causal paths of an action are not present in the person’s consciousness, and the experience of conscious will arises as we infer this path from our thought to our action (Wegner, 2002a, b). According to his theory, we unconsciously decide upon an outcome, and if this decision coincides with our conscious intention, we experience having made this choice independent of the unconscious processing. This phenomenon appears to be what happened in the implicit choice condition: most participants used an automatic behaviour influenced by external factors (position and reachability effects), but were not consciously aware of these influenced, underestimating the number of people who would have chosen the same card as they did.

### Feeling of freedom

We also measured participants’ general sense of freedom for their choice (Experiment 1 and 2) as well as its three components (Experiment 2) according to Thompson’s definition (Thompson et al., 1990). Participants’ feelings of deliberation, restriction, and control for their choice were measured, alongside their general feeling of freedom. Regarding the general sense of freedom across both experiments, participants’ choice of card did not have any significant impact. This shows that whether people were influenced by the force or not, they felt the same degree of freedom for their choice. As Binet already noted (Binet, 1894), “each individual placed in certain conditions, and thinking to be acting freely, is, in reality, behaving in the same way as other individuals, and what they have in common is automatic activity” (p. 151). This adds to previous results regarding people’s awareness of their bias, and support the choice blindness literature, showing that people tend to be blind to the reasons for their choice (Hall et al., 2010; Johansson, Hall, Sikström, Tärning, & Lind, 2006; Rieznik et al., 2017).

However, participants felt their choice was more deliberate when they did not choose the target card. The feeling of deliberation was the only component which was not correlated with the general sense of freedom or its other two components. During the debriefing, participants who did not choose the target card typically reported first thinking about taking it and then changing their mind for another one. This suggests that people can be aware of their metacognition about their choice, while still being blind to why they are acting in the way they do.

### Towards freer choices?

Our results highlight important new pathways to explore the nature of free will. If people can become aware of their metacognition about their decisions, they can inhibit their initial impulsive and automatic behaviour and decide not to act upon them. Baumeister, notes that one needs to go through an inner process of choosing for free will to be relevant. He describes how the role of free will would be to alter the flow of our behaviour, and how “the capacity for rational thought and decision-making lies atop an irrational, impulsive beast, and so it only sometimes can alter the cause of action that that impulsive beast will take” (p. 71).

Dovetailing this idea, our results suggest that we should refocus the debate on determinism vs. free will and frame the latter in terms of degrees. Baumeister linked free will and dual-process theories of human mental functioning by pointing out that free will could be mainly associated with what is called System 2, or the cognitive processes involving conscious and controlled activity rather than the nonconscious and automatic one associated with System 1 (2002). Investigating freedom in terms of autoregulation and self-control might help us find ways to conquer these degrees of freedom of choice. Such empirical findings may help us understand the mechanisms that underpin our reasoning and help us make more deliberate choices, rather than simply acting on habits and automatic behaviours. This research may help us find concrete and practical ways to enhance our deliberate and rational cognitive processes. Our paper suggests that simply making people more aware that they are making a decision could be one efficient solution.

## Footnotes

1 Participants who used their right hand to had a significantly higher handedness score (H(1) = 12.67, *p* < .001), which illustrates that the selection as typically made with the dominant hand.
